# Gingival crevicular fluid biomarkers in type 1 diabetes mellitus: A case–control study

**DOI:** 10.1002/cre2.351

**Published:** 2020-12-24

**Authors:** Maria Sereti, Margaux Roy, Alkisti Zekeridou, Giacomo Gastaldi, Catherine Giannopoulou

**Affiliations:** ^1^ Division of Regenerative Dentistry and Periodontology University Clinics of Dental Medicine, University of Geneva Geneva Switzerland; ^2^ Diabetology Unit University Hospitals of Geneva Geneva Switzerland

**Keywords:** AGEs, gingival crevicular fluid, IL‐8, MMP‐8, periodontal disease, type 1 diabetes

## Abstract

**Objective:**

The aim of the study is to compare the levels of Gingival Crevicular Fluid (GCF) interleukin 8 (IL‐8), matrix metalloproteinase 8 (MMP‐8) and advanced glycated‐end products (AGEs) in a cohort of type 1 diabetic (T1D) subjects and healthy controls.

**Material and methods:**

GCF samples and periodontal examination were assessed in 50 subjects with T1D (30 males and 20 females; mean age: 35.2 years) recruited from the Diabetology Unit of the Geneva University Hospitals and in 50 control subjects matched for gender, age and smoking status. Samples were assessed for IL‐8 and MMP‐8 using a bead array multianalyte detection system and for AGEs the ELISA. The two groups were compared using the Wilcoxon signed rank test.

**Results:**

The mean HbA1c differed significantly between the groups (8.3% for the T1D group vs. 5.2% for the control group, *p* < 0.001). T1D subjects had significantly more plaque and gingival inflammation and presented more sites with bleeding on probing compared to the controls. The GCF levels of IL‐8, MMP‐8 and AGEs did not differ significantly between the groups. Further analysis of the GCF markers in younger (<40 years) and older (≥40 years) cohorts, revealed no significant differences between younger diabetics and controls or between older diabetics and controls. When the groups were divided according to their glycemic status (HbA1c 6.1–8, and > 8%), again no significant differences could be identified for any of the biochemical markers.

**Conclusions:**

T1D subjects, particularly the younger ones, exhibited more inflammation compared to the matched healthy controls. Results on the GCF expression of IL‐8, MMP‐8 and AGEs did not differ between the groups. The diabetic population of our cohort was for the most part fairly‐controlled, with little if any complications and with presence of only mild type of periodontal disease, as 68% had gingivitis.

## 1 INTRODUCTION

Type 1 diabetes is a chronic autoimmune disease characterized by insulin deficiency as a consequence of the insulin producing pancreatic islet cells destruction (DiMeglio, Evans‐Molina, & Oram, 2018). Several genetic, environmental and immunologic factors contribute to the pathogenesis of T1D (Paschou, Papadopoulou‐Marketou, Chrousos, & Kanaka‐Gantenbein, 2018). The onset of the disease is not limited to childhood, as several studies have reported that 50% of the cases can occur in adulthood (Nicholas J. Thomas et al., 2018; N. J. Thomas et al., 2019). The incidence and prevalence of T1D are increasing continuously. In fact, there is an overall annual increase in incidence of about 2–3% per year (Mayer‐Davis et al., 2017).

A bidirectional association has been documented between periodontitis and type 2 diabetes (T2D): diabetes is correlated with an acceleration of periodontal destruction and vice versa, periodontitis can affect the glycemic control (Chavarry, Vettore, Sansone, & Sheiham, 2009; Graves, Ding, & Yang, 2020; Lalla & Papapanou, 2011)^,^. The link between the two conditions is attributed to the established hyperglycemia which leads to increased levels of reactive oxygen species, cytokines and advanced glycation end products (AGEs) in the periodontium (Abbass, Korany, Salama, Dmytryk, & Safiejko‐Mroczka, 2012; Graves et al., 2020; Lalla, Lamster, Drury, Fu, & Schmidt, 2000).

Less studies have examined interactions between T1D and periodontal health, yet a recent systematic review found a prevalence of periodontitis in T1D patients to be 18.5% (Dicembrini et al., 2020).

The Gingival Crevicular Fluid (GCF) is a serum exudate found in the sulcus (McCulloch & 1994) consisting of both serum and locally produced products (Armitage, 2004; Kinney et al., 2014). More specifically, in the GCF we can detect components of three main categories: host‐derived enzymes and their inhibitors, inflammatory mediators and host‐response modifiers as well as tissue breakdown products (Armitage, 2004). Moreover, the sampling of the GCF is an easy, non‐invasive technique and offers the possibility to examine various sites simultaneously from the same subject.

In some of these studies, the analysis of GCF was used as a tool to investigate whether T1D modulates the local levels of several inflammatory biomarkers when associated to gingivitis or periodontitis (Akcalı et al., 2017; Aral, Nalbantoğlu, Nur, Altunsoy, & Aral, 2017; Safkan‐Seppala, Sorsa, Tervahartiala, Beklen, & Konttinen, 2006; Salvi et al., 2010).

Among a plethora of markers, matrix metalloproteinase‐8 (MMP‐8), also known as collagenase‐2 or neutrophil collagenase and member of the MMP family, has been extensively studied in oral fluids (Al‐Majid et al., 2018). MMP‐8 is the main collagenase that is found in inflamed gingiva in adult periodontitis and is associated with pathologic extracellular matrix destruction (Sorsa, Uitto, Suomalainen, Vauhkonen, & Lindy, 1988). A recent review based on 61 articles reported that active MMP‐8 has the potential ‐ alone or in combination with other pro inflammatory markers and/or microbiological markers ‐ to serve as an adjunctive diagnostic tool in periodontal and periimplant disease (Al‐Majid et al., 2018).

Interleukin‐8 (IL‐8) has been identified as a neutrophil chemotactic factor, produced by various types of cells upon stimulation and inducing neutrophils to release lysosomal enzymes. The levels of IL‐8 in the GCF were found to be significantly higher in chronic periodontitis patients compared to controls while they decreased after periodontal treatment (Konopka, Pietrzak, & Brzezińska‐Błaszczyk, 2012). Concerning the role of IL‐8 in relation to the T2D‐associated periodontal destruction, contradictory results have been reported (Javed, Al‐Askar, & Al‐Hezaimi, 2012).

Chronic hyperglycemia is related to the enhanced production of AGEs. The formation of irreversible AGEs affects the tissues by compromising the physiologic and mechanical functions, as a result of defective constitution of the extracellular matrix (ECM) components (Gurav, 2013). With regards to periodontal tissues, in vitro studies have indicated that AGEs are implicated in suppressed collagen production by gingival and periodontal ligament fibroblasts (Murillo et al., 2008; Ren, Fu, Deng, Qi, & Jin, 2009). Moreover, a clinical study on the immunohistochemical expression of AGEs in biopsies of gingival tissues found that T1D subjects presented a significantly higher percentage of AGE‐positive cells than did T2D subjects, not only in the epithelium, but also in vessels and fibroblasts (Zizzi et al., 2013). A possible mediator for these processes could be the cell surface receptor for AGE products and its ligands that are expressed in the periodontium of individuals with Diabetes Mellitus (DM) (Lalla & Papapanou, 2011).

We have recently shown that patients with T1D presented more plaque and more inflammation than healthy controls, particularly the younger subjects (Roy, Gastaldi, Courvoisier, Mombelli, & Giannopoulou, 2019). Our hypothesis was that the accumulation of bacterial biofilms in T1D subjects may lead to more severe gingivitis, which in turn may increase the risk to develop periodontitis. In this context, we further hypothesized that in diabetic subjects, the levels of a number of locally produced inflammatory markers would increase as compared to non‐diabetic individuals, corroborating the clinical periodontal status. Thus, by using the same cohort, the objective of the present study is to evaluate the GCF levels of MMP‐8, IL‐8 and AGEs in T1D patients with different glycemic levels and to compare them to healthy controls.

## 2 MATERIAL AND METHODS

### 2.1 Study design

Fifty subjects with diagnosis of T1D were recruited from the patient cohort of the Diabetology Unit of the Geneva University Hospital. Subjects were aged between 18 and 85 years old, presented at least 10 natural teeth and were diagnosed for T1D for more than 1 year. The control group consisted of 50 systemically healthy subjects matched for age, sex and smoking status and were recruited among patients attending the University Clinics of Dental Medicine of the University of Geneva. The protocol was approved by the Ethical Committee of the University Hospitals of Geneva and the study was conducted according to the principles outlined in the Declaration of Helsinki on human medical experimentation. Written informed consent was obtained from all participants.

### 2.2 Medical visit and periodontal examination

The medical and periodontal examination visit have been previously described in detail (Roy et al., 2019). In brief, during the routine medical visit of the subjects at the Diabetology Unit, the following data were compiled: date of diagnosis, presence of specific antibodies related to T1D, mean glycated hemoglobin in the past 3 years, body mass index, number of diabetes complications (retinopathy, nephropathy, neuropathy and macrovascular complications), kind of glycose monitoring and insulin route of administration.

The participants of the control group were submitted to a quick HbA1c test in order to confirm the absence of diabetes.

The dental visit consisted of a full‐mouth clinical examination at six sites per tooth and included the evaluation of Plaque Index (PI) (Silness & Löe, 1964), Gingival Index (GI) (Löe & Silness, 1963), Probing Pocket Depth (PD), Bleeding on probing (BOP), Gingival Recession (REC), furcation involvement and tooth mobility. Attachment Loss (AL) was calculated based on the PD and REC values and we used the CDC/AAP classification to evaluate the severity of periodontitis (Albandar, 2007). No radiographic assessment was performed.

### 2.3 Gingival crevicular fluid collection

GCF was collected after the evaluation of Plaque Index and before all other periodontal parameters. GCF was collected by means of Durapore membrane strips (2 × 6mm, 0.22 μm pore size; Millipore, Bedford, MA) at the mesial‐buccal aspect of two teeth, that is, at one site in two dentition quadrants. If available, the first molar was chosen. In case of absence of this tooth, the second molar, second premolar or the first premolar was chosen. First, supragingival plaque was carefully removed with cotton pellets and the sites were gently dried with an aspiration tip. After waiting 2 min, the strips were placed at the entrance of the selected sulcus or pocket for 1 min and then transferred into microtube. The two samples were pooled and stored at −80°C until analyzed. Samples contaminated with blood were discarded.

### 2.4 Laboratory procedures

The day of the analysis each GCF sample was eluted in 80 μl PBS, vortexed for 1 min and then centrifuged for 10 min at 13,000 rpm. Aliquots of 25 μl and 50 μl of each GCF sample were used in order to run in parallel 2 assays, one for the cytokine IL‐8 and the MMP‐8, and one for the AGEs. The assays were performed in 96‐well filter plates following the manufacturer's instructions. We used the 2‐plex fluorescent bead‐based immunoassay (Miliplex Map Kit, Human Sepsis Panel Magnetic Bead Panel, cat. #HSP2MAG‐63 K) and the Bio‐Plex 200 suspension array system (Bio‐Rad laboratories, Hercules, CA) for the detection of MMP‐8 and IL‐8 as well as the AGEs ELISA kit (OxiSelect Advanced Glycation End Product (AGE)). Competitive ELISA Kit for the detection of AGEs. IL‐8 data were reported as pg/ml, AGEs and MMP‐8 data as μg/ml. A constant (0.1) was added to all readings to remove zero values.

### 2.5 Statistical analysis

Diabetic and non‐diabetic participants were described using frequencies and percentages for categorical variables and mean and standard deviations for continuous variables. We used the Mann–Whitney‐*U* test to compare diabetic versus non‐diabetic subjects. Results on the levels of biochemical markers were expressed as median and first and third quartile. We used the same test for comparisons when each group (diabetic, non‐diabetic) was further divided in younger (<40 years old) and older (≥40 years age) and when separating the diabetic group according to HbA1c levels. For the T1D group, we ran linear regression analysis to reveal associations between the levels of each biochemical parameter (primary outcome) and several factors, such as duration of diabetes in years, diabetes control (HbA1c), levels of other biomarkers in GCF and the various clinical parameters as predictors. For the same group, we further used multiple linear regression analysis to reveal associations between the levels of HbA1c and several factors such as age, diabetes duration and the levels of AGEs, IL‐8 and MMP‐8 and we used the Pearson's correlation test to assess correlations between the levels of HbA1c and the various periodontal parameters (Plaque Index, Gingival Index, Probing depth and BOP). All analyses were conducted using R v3.5.1, with a significance threshold set at *p* < 0.05, except for the multiple regression where the significance threshold was at *p* < 0.02.

## 3 RESULTS

Fifty subjects with type I diabetes mellitus and 50 non‐diabetic subjects matched for gender, age and smoking status participated in the study. The characteristics of the study population have been described in our previous paper (Roy et al., 2019). Briefly, the mean age was 35 years and approximately 40% of the subjects of both groups were females. One subject in the diabetic group was younger than his matched control, thus the difference appears as significant in the table (Table 1). The 3 years mean HbA1c in the diabetic group was 8.3% and in the non‐diabetic group 5.2%, the difference being statistically significant (*p* < 0.001). The mean time since diagnosis of diabetes was 13.3 years (±11.9) and the mean number of complications per subject was 0.6. T1D subjects had a mean BMI of 24.5 (±3.8) (Table 1).

**TABLE 1:** Characteristics of the study population

	Control	Diabetic	*p*
Gender			1.000
Male	30 (60)	30 (60)	
Female	20 (40)	20 (40)	
Age (years), mean ± SD	35.9 (15.0)	35.2 (15.0)	**0.020**
HbA1c (%), mean ± SD	5.2 (0.4)	8.3 (1.8)	**<0.001**
Duration of diabetes (years), mean ± SD		13.3 (11.9)	
Number of complications, mean ± SD		0.6 (1.0)	

*Note*: The significance level for bold value is p < 0.05.

As previously reported (Roy et al., 2019), in the control group, 12% of the subjects had a healthy periodontium, 60% gingivitis, 14% a mild form of periodontitis, 4% a moderate form and 10% a severe form. In the diabetic group, 2% had a healthy periodontium, 68% gingivitis, 12% a mild form of periodontitis, 10% a moderate form and 8% a severe form. No statistically significant difference was found between the groups. As shown in Table 2, several parameters, such as the number of teeth, the mean PD, REC, AL and the mean number of sites with PD > 4 mm that bled upon probing did not differ between the groups. The mean presence of plaque, GI, BOP and the mean number of sites with PI and GI score ≥ 1, were significantly higher in the diabetic as compared to non‐diabetic group. As shown in the same table, the median GCF levels of IL‐8 (control: 225 pg/ml vs. diabetic: 220 pg/ml, *p* = 0.433), MMP‐8 (control: 38.3 μg/ml vs. diabetic: 32.1 μg/ml, *p* = 0.538), and AGEs (control: 5.8 μg/ml vs. diabetic: 3.4 μg/ml, *p* = 0.905), did not differ significantly between the groups. However, a wide variation was observed.

**TABLE 2:** Dental and biochemical parameters of the study groups

	Control	Diabetic	*p*
Number of teeth, mean ± SD	26.2 (2.8)	26.8 (2.6)	0.248
PI, mean ± SD	0.4 (0.2)	0.5 (0.4)	**0.014**
GI, mean ± SD	0.4 (0.4)	1.1 (0.7)	**<0.001**
BOP, mean ± SD	29.4 (16.4)	40.5 (22.2)	**0.009**
PD, mean ± SD	2.5 (0.3)	2.5 (0.4)	0.381
Recession, mean ± SD	0.2 (0.2)	0.3 (0.3)	0.083
AL, mean ± SD	2.6 (0.4)	2.8 (0.6)	0.070
Number of sites PI>1, mean ± SD	13.8 (14.5)	23.9 (27.2)	**0.047**
Number of sites GI > 1, mean ± SD	18.8 (23.1)	59.2 (57.6)	**0.001**
Number of sites PD > 4 + BOP	1.5 (3.7)	2.3 (5.0)	0.336
AGEs in μg/ml, median (Q1, Q3)	5.8 (0.9, 13.1)	3.4 (0.7, 47.6)	0.905
MMP‐8 in μg/ml, median (Q1, Q3)	38.3 (15.7, 67.8)	32.1 (17.8, 55.1)	0.538
IL‐8 in pg/ml, median (Q1, Q3)	225 (150, 414)	220 (145, 331)	0.433

*Note*: The significance level for bold value is p < 0.05.

We further compared the levels of the three biochemical markers between controls and diabetics, in younger (<40 years old) and older (≥40 years old) subjects. We had previously shown that diabetics <40 years old had significantly more plaque and more inflammation (GI) compared to their matched controls. In the older group (≥40 years old), only gingival inflammation was significantly higher in diabetics compared to controls. As shown in Table 3, these differences were not reflected in the levels of the GCF markers, as no significant differences were observed between younger diabetics and controls, as well as between older diabetics and controls.

**TABLE 3:** Biochemical parameters in controls and diabetics <40 years and ≥40 years old

	<40 years old		*p*	≥40 years old		*p*
	Control (n = 28)	Diabetic (n = 29)		Control (n = 22)	Diabetic (n = 21)	
AGEs in μg/ml, median (Q1, Q3)	6.5 (1.3, 12.9)	1.8 (0.6, 12.9)	0.137	4 (0.4, 29.1)	13.3 (0.6, 116.9)	0.276
MMP‐8 in μg/ml, median (Q1, Q3)	38.2 (15.1, 65.4)	30.1 (13.2, 51.4)	0.568	40.5 (20.2, 80.1)	34.3 (21.7, 65.1)	0.683
IL‐8 in pg/ml, median (Q1, Q3)	226 (170, 483)	223 (109, 311)	0.213	222 (131, 332)	219 (146, 383)	0.699

Then, we divided the diabetic group in two sub‐groups according to their level of HbA1c: group 1 included 27 subjects with an HbA1c level of 6.1%–8%; group 2 included 23 subjects with HbA1c level of >8% and control group included the 50 control subjects. As shown in Table 4, the clinical parameters differed between the two diabetic groups and the controls, however, no differences were observed between the diabetic sub‐ groups and the controls, on the level of the studied biochemical markers. For the diabetic group, regression analysis between each GCF marker and a variety of factors such as levels of HbA1c, diabetes duration and periodontal parameters, revealed a significant association between the levels of MMP‐8 and IL‐8 (*p* < 0.001) and between the levels of AGEs and PD (*p* < 0.004) and only marginally with GI (*p* < 0.055) (Appendix Table).

**TABLE 4:** Clinical and biochemical parameters among patients in groups 1, 2 and control

Parameters	Control *N* = 50	Group 1 N = 27	*p* ^a^	Group 2 N = 23	*p* ^b^
Plaque index, mean ± SD	0.4 (0.2)	0.5 (0.4)	**0.008**	0.6 (0.5)	**0.002**
Gingival index, mean ± SD	0.4 (0.4)	1.0 (0.5)	**<0.001**	1.1 (0.9)	**<0.001**
BOP, mean ± SD	29.4 (16.4)	39.1 (19.7)	**0.02**	37.0 (25.8)	**0.05**
MMP‐8 μg/ml, median (Q1, Q3)	38.3 (15.7, 67.8)	58.2 (26.2, 83.2)	0.685	31.5 (15.1, 56.1)	0.541
IL‐8, pg/ml, median (Q1, Q3)	225 (150, 414)	267 (172, 458)	0.529	217 (144, 402)	0.517
AGEs, μg/ml, median (Q1, Q3)	5.8 (0.9, 13.1)	9.9 (0.9, 57.9)	0.873	2.5 (0.9, 11.3)	0.980

*Note*: The significance level for bold value is p < 0.05. Group 1: Diabetics with HbA1c: 6.1–8%; Group 2: Diabetics with HbA1c >8%; Control: Non‐diabetics.

^a^ Comparison between control and group 1.

^b^ Comparison between control and group 2.

For the same group, the level of HbA1c was marginally and in a negative manner associated to the subject's age (*p* = 0.003). Furthermore, when the Pearson's correlation test was used to assess correlations between the levels of HbA1c and the various periodontal parameters (Plaque Index, Gingival Index, Probing depth and BOP), no correlation was observed (data not shown).

## 4 DISCUSSION

The aim of the present study was to evaluate the levels of three biomarkers in the GCF of T1D patients and compare them to a group of matched healthy controls. The periodontal health conditions and oral health behavior of the cohorts have been reported in detail in our previous paper (Roy et al., 2019). The results showed similar levels of IL‐8, MMP‐8 and AGEs between T1D and healthy patients. However, broad variations were found on the levels of all three biomarkers.

Several studies have examined the prevalence of periodontal pathogens and systemic and /or local inflammatory mediators in T1D subjects and healthy controls either in GCF or serum samples. Duque et al. (Duque et al., 2017), reported higher lipid parameters in the T1D group compared to the control group but similar levels of both the “red complex” bacteria and of IL‐1β, TNF‐α and IL‐6 found in the serum. On the contrary, do Nascimento et al. (do Nascimento de Oliveira et al., 2018) using a proteomic approach for the analysis of serum samples, reported a differential expression of eight proteins between TD1 and control subjects.

In our study, similarly to many others, we used the GCF as a tool to analyse the locally derived biomarkers related to periodontal inflammation (IL‐8, MMP‐8) and diabetes (AGEs). The GCF was not collected in pre‐selected sites and the analysis of the biochemical markers was assessed on pooled samples for each patient. Thus, we cannot rule out the possibility that if GCF was collected in pre‐determined “periodontally affected” sites in each subject, pronounced differences in the GCF levels of the three biomarkers would have been detected. However, our group has recently shown that GCF analysis can discriminate patients with periodontal disease but cannot discriminate between periodontally healthy and diseased sites in the same patient (Zekeridou, Giannopoulou, Cancela, Courvoisier, & Mombelli, 2017).

In one study, including T1D with T2D subjects with periodontitis, it was found that the GCF levels of IL‐1β and TNF‐α were significantly higher in the T1D subjects as compared to the T2D subjects and these levels were negatively affected by the duration of the diabetes (Aspriello et al., 2011). However, no comparison was made between T1D and healthy subjects. Salvi et al. (Salvi, Kandylaki, Troendle, Persson, & Lang, 2005) during a period of experimental gingivitis in subjects with and without T1D, observed a higher and quicker inflammatory response in the T1D group while a secondary analysis of the same group (Salvi et al., 2010) showed significantly higher GCF levels of Il‐1β and MMP‐8 for the T1D subjects. Compared to this study, our cohort did not refrain from normal dental hygiene, as it was the case with the experimental gingivitis model which resulted in a much greater plaque index score and a more pronounced inflammatory state.

Recently, a systematic review concluded that T1D is an important risk factor for periodontitis and that the severity of periodontitis seems to vary widely between patients with optimal (HbA1c ≤7%) and suboptimal (HbA1c >7%) glycaemic control (Dicembrini et al., 2020). As proposed by Jindal et al. (Jindal, Singh Parihar, Sood, Singh, & Singh, 2015), we chose a cut‐off value of 8 for the levels of HbA1c in order to assess whether a “fair” or poor glycemic control is related to the biomarkers' levels. Although the clinical parameters, in terms of gingival inflammation, were worse in these two groups as compared to the control group, the biochemical analyses failed to show any differences. It should be pointed out, however that our diabetic group had stable HbA1c levels the last 3 years, had a relatively low BMI score (24.5 ± 3.8), presented very few complications and was followed in diabetes specialty practices. In fact, the complications related to the diabetes were mainly retinopathy (11 subjects), nephropathy (7 subjects), neuropathy (7 subjects) and only one subject presented a major complication affecting the microvasculature (cardiovascular disease). As for dental health, subjects had good oral hygiene and attended regularly the dental office.

A study by Lalla et al. (Lalla et al., 2006) examined subgingival plaque bacteria samples and IgG serum levels between T1D with a stable HbA1c profile and healthy patients. In accordance with our study, they found no significant differences after controlling for the severity of periodontal disease between the two groups. The authors concluded that the severity of periodontal disease determines the infection profiles, and that in similar clinical situations, the serum antibody responses do not differ significantly in the presence of T1D. Similarly, the lack of differences in the biomarker levels in our analyses, could be attributed to the fact that the majority of our subjects had gingivitis and not severe periodontitis. This hypothesis is also supported by another study comparing the collagenases' levels in the GCF of T1D patients with periodontitis versus healthy subjects, which found no difference between the diabetic well‐controlled group and the healthy group (Safkan‐Seppala et al., 2006). Furthermore, the poor controlled group demonstrated worse clinical parameters and higher collagenase activity as well, when compared to the well‐controlled and healthy group. These results, together with ours, suggest that in a diabetic population that is regularly followed‐up by a specialist, in a way that a stable level of HbA1c is achieved and without diabetic complications, the changes in the inflammatory markers in the GCF may be minimal, even in the presence of periodontal disease.

We further found that the levels of HbA1c in the diabetic group, although marginally, were negatively associated to the subjects' age. Recent data collected on 16′061 individuals with T1D and aged between <4 and >75 years old, showed that HbA1c levels can vary considerably with age, with higher levels during childhood and adolescence, a gradual decline until ~30 years of age followed by a plateau, and again a modest drop after 65 years of age (Miller et al., 2015).

It was surprising that the levels of AGEs did not differ significantly between the two groups. To our knowledge, only few studies exist on the levels of AGEs in the GCF. Most of them have analyzed saliva samples (Uh et al., 2004) where the concentration of AGEs is often higher than that in the GCF. A study conducted on patients with and without periodontitis as well as with and without T2D, found significantly higher GCF levels of AGEs in the diabetic group with periodontitis as compared to the non‐diabetics, independently of the presence or not of periodontitis (Akram, Alqahtani, Alqahtani, Al‐Kheraif, & Javed, 2020). Comparison with our study is difficult as the populations differed not only on the type of diabetes but also on their BMI score and on their periodontal status. Zizzi et al. (Zizzi et al., 2013) examined gingival biopsies of T1D and T2D patients with periodontitis versus healthy controls and found a significant correlation of AGEs levels with the duration of diabetes, while no correlation was found with the HbA1c levels. Until now, only one study examined the levels of AGEs in relation to different glycemic levels and the correlations with clinical parameters. Although the study included T2D subjects and the levels of AGEs were studied in the peri‐implant sulcular fluid (PISF), it was found that the mean levels of AGEs in PISF were increased in those individuals with high glycemic levels (Al‐Sowygh, Ghani, Sergis, Vohra, & Akram, 2018).

The main limitation of our study is that our cohort was probably not representative of all T1D subjects as those with more severe complications related to diabetes, having an irregular follow‐up for their diabetes, being uninsured and having severe periodontitis were not represented. As the clinical examination was performed after the GCF sampling procedure, we could not assess both healthy and periodontally diseased sites from the same patient.

Furthermore, radiographic evaluation was not assessed, thus the diagnosis of periodontal disease was not based in the new classification system.

In conclusion, the present study showed similar levels of MMP‐8, IL‐8 and AGEs in the GCF of T1D and healthy subjects, despite the fact that the diabetic cohort presented slightly higher prevalence of gingivitis (68% vs. 60%). The diabetic cohort for the most part, consisted of fair‐controlled patients that presented very low level of complications and presence of only mild type of periodontal disease. Thus, at this stage of periodontal inflammation and probably of diabetes severity, differences in the biomarker levels in the GCF cannot yet be yielded. Longitudinal studies and comparisons between groups with different levels of periodontal inflammation and/or poorly versus well controlled T1D status are needed to better understand the site‐specific periodontal destruction among these subjects.

## CONFLICT OF INTEREST

The authors declare no conflict of interest.

## Data Availability

The data that support the findings of this study are openly available in [repository name e.g “figshare”] at http://doi.org/[doi], reference number [reference number].

## Appendix

**Table:**

Model	Unstandardized coefficients B	Stand. Error	Standardized coefficients B	*t*	Sign.
(constant)	257.058	234.160		1.098	0.279
AGEs	−0.002	0.011	−0.032	−0.203	0.840
HbA1c	−14.488	15.910	−0.130	−0.911	0.368
yearsDiab	−0.918	2.392	−0.062	−0.384	0.703
MMP8	0.003	0.001	0.532	3.544	0.001
PI	−88.346	100.865	−0.178	−0.876	0.387
GI	−10.690	78.986	−0.038	−0.135	0.893
PD	43.277	86.122	0.094	0.503	0.618
BOP	−66.473	157.438	−0.081	−0.422	0.675

*Note*: Dependent Variable: IL‐8.

**Table:**

Model	Unstandardized coefficients B	Stand. Error	Standardized coefficients B	*t*	Sign.
(constant)	−42,810.446	41,873.942		−1.022	0.313
AGEs	1.219	1.906	0.094	0.639	0.526
HbA1c	584.906	2,868.612	0.028	0.204	0.840
yearsDiab	464.602	420.995	0.165	1.104	0.277
IL8	88.941	25.099	0.467	3.544	**0.001**
PI	13,700.256	18,044.905	0.145	0.759	0.452
GI	−8,713.012	14,028.342	−0.161	−0.621	0.538
PD	12,973.623	15,276.046	0.148	0.849	0.401
BOP	44,731.729	27,211.377	0.285	1.644	0.108

*Note*: Dependent Variable: MMP‐8.

**Table:**

Model	Unstandardized coefficients B	Stand. Error	Standardized coefficients B	*t*	Sign.
(constant)	−8,256.572	3,334.721		−2.476	0.018
MMP8	0.009	0.014	0.113	0.639	0.526
HbA1c	272.375	238.951	0.166	1.140	0.261
yearsDiab	−61.219	34.821	−0.281	−1.758	0.087
IL8	−0.497	2.450	−0.034	−0.203	0.840
PI	2,190.055	1,497.742	0.299	1.462	0.152
GI	−2,249.433	1,136.571	−0.537	−1.979	**0.055**
PD	3,623.519	1,165.736	0.534	3.108	**0.004**
BOP	−1,041.271	2,378.349	−0.086	−0.438	0.664

*Note*: Dependent Variable: AGEs.
